# Insights into Ongoing Evolution of the Hexachlorocyclohexane Catabolic Pathway from Comparative Genomics of Ten Sphingomonadaceae Strains

**DOI:** 10.1534/g3.114.015933

**Published:** 2015-04-07

**Authors:** Stephen L. Pearce, John G. Oakeshott, Gunjan Pandey

**Affiliations:** CSIRO Ecosystem Sciences, Acton, ACT-2601, Australia

**Keywords:** IS6100, evolution, hexachlorocyclohexane, lindane, transposon

## Abstract

Hexachlorocyclohexane (HCH), a synthetic organochloride, was first used as a broad-acre insecticide in the 1940s, and many HCH-degrading bacterial strains have been isolated from around the globe during the last 20 years. To date, the same degradation pathway (the *lin* pathway) has been implicated in all strains characterized, although the pathway has only been characterized intensively in two strains and for only a single HCH isomer. To further elucidate the evolution of the *lin* pathway, we have biochemically and genetically characterized three HCH-degrading strains from the Czech Republic and compared the genomes of these and seven other HCH-degrading bacterial strains. The three new strains each yielded a distinct set of metabolites during their degradation of HCH isomers. Variable assembly of the pathway is a common feature across the 10 genomes, eight of which (including all three Czech strains) were either missing key *lin* genes or containing duplicate copies of upstream *lin* genes (*linA-F*). The analysis also confirmed the important role of horizontal transfer mediated by insertion sequence IS6100 in the acquisition of the pathway, with a stronger association of IS6100 to the *lin* genes in the new strains. In one strain, a *linA* variant was identified that likely caused a novel degradation phenotype involving a shift in isomer preference. This study identifies a number of strains that are in the early stages of *lin* pathway acquisition and shows that the state of the pathway can explain the degradation patterns observed.

Hexachlorocyclohexane (HCH) is a synthetic organochloride that saw widespread global use after the discovery of its insecticidal properties in the 1940s. However, the off-target toxicity of HCH has caused it to be phased out of use during the last two decades in many countries and it was added to the Stockholm Convention list of persistent organic pollutants in 2009 (Vijgen *et al.* 2011). The manufacture of HCH produces a number of different isomers that differ in the equatorial and axial arrangement of hydrogen and chlorine atoms. Only one isomer (γ-HCH, known commercially as lindane) has useful insecticidal properties, and this isomer makes up only a small proportion (8–15%) of the HCH produced (Breivik *et al.* 1999). The remaining isomers (predominantly α-HCH, 55–80%, β-HCH, 5–14%, and δ-HCH, 2–16%), most of which still show high mammalian toxicity (Breivik *et al.* 1999), are separated and dumped in large waste stockpiles around the world (Lal *et al.* 2010). The long half-life (*e.g.*, 1.6 and 4.8 years for α- and β-HCH, respectively, in soil) of many HCH isomers means that they will remain an environmental hazard for many years (Wöhrnschimmel *et al.* 2012).

More than 60 HCH-degrading bacterial strains have been isolated from contaminated fields since the first such strain was reported in 1989 (Johri *et al.* 1998; Senoo and Wada 1989; Thomas *et al.* 1996). These strains are predominantly from the family Sphingomonadaceae; however, strains from distant genera such as *Microbacterium* and *Bacillus* also are represented (Lal *et al.* 2010). The most complete understanding of bacterial HCH degradation to date concerns the study of the insecticidal γ-HCH isomer. Interestingly, only one pathway (mediated by the *lin* genes) has currently been identified for its degradation, with examples found both inside and outside the Sphingomonadaceae. Many aspects of the pathway are still unclear; however, two recent reviews extensively cover our current understanding (Lal *et al.* 2010; Nagata *et al.* 2007). The *lin* pathway was originally identified in *Sphingobium japonicum* UT26 and is composed of an upstream [conversion of HCH to 2,5-dichlorohydroquinone (2,5-DCHQ)] and downstream component (conversion of 2,5-DCHQ into tricarboxylic acid cycle intermediates). The upstream pathway initially proceeds via two successive dehydrochlorinations (mediated by LinA) followed by two LinB-catalyzed hydrolytic dechlorinations and a dehydrogenation by LinC to produce 2,5-DCHQ (Nagata *et al.* 1993a,b; Nagata *et al.* 1994). The downstream pathway involves an initial reductive dechlorination by the glutathione *S*-transferase LinD, followed by ring cleavage and conversion to β-ketoadipate by LinE and LinF, respectively (Miyauchi *et al.* 1999, 1998). β-Ketoadipate is a common intermediate in aromatic metabolism and is converted into tricarboxylic acid cycle intermediates by LinGH and LinJ (Nagata *et al.* 2007).

The catabolic pathway for the major isomers other than γ-HCH has not been fully elucidated; however, *lin* genes are known to play a role. The initial steps in the degradation of these other isomers can be catalyzed by either LinA or LinB, depending on the axial and equatorial arrangement of chlorine atoms in each isomer. β-HCH lacks the 1,2-biaxial HCl pair required by the reaction mechanism of LinA, and is instead initially metabolized by LinB (Trantirek *et al.* 2001). α- and δ-HCH are each capable of degradation by LinA *in vitro*, but in bacterial cell cultures δ-HCH appears to be initially degraded by LinB (Geueke *et al.* 2013; Kumar *et al.* 2005; Suar *et al.* 2005; Wu *et al.* 2007). The expression ratio of LinA to LinB is known to affect the initial metabolism of HCH isomers and is the likely explanation for why δ-HCH is degraded via LinB (Geueke *et al.* 2013). The subsequent steps in degradation of these three isomers are as yet unknown. A summary of the degradation pathway for each isomer relevant to this work can be found in Supporting Information, Figure S1.

As a recently introduced synthetic compound, HCH also provides a model system for examining how bacteria evolve new catabolic pathways. Although they are chromosomally located in *S. japonicum* UT26, most upstream components of the *lin* pathway are found in unique regions of the UT26 genome associated with the insertion sequence IS*6100*, suggesting that they have been acquired through horizontal transfer (Lal *et al.* 2006; Nagata *et al.* 2011). IS*6100* is a member of the IS6 family, which can mobilize genes between two directly repeated IS*6100* elements through replicon fusion and subsequent resolution by homologous recombination (Mahillon and Chandler 1998). This process results in an increase in the number of copies of IS*6100* in a genome, with a copy of the element left at the original site.

The horizontal transfer of *lin* genes is observed in other strains, such as the recently sequenced *Sphingomonas* sp. MM-1, in which all the upstream *lin* genes are found on plasmids (Tabata *et al.* 2011; Tabata *et al.* 2013). A number of other genomes of HCH-degrading *Sphingobium* strains isolated from India and Japan have been sequenced recently, although their genomic organization of *lin* genes is yet to be fully described (Kohli *et al.* 2013; Kumar Singh *et al.* 2013; Mukherjee *et al.* 2013). In addition to these isolated strains, the metagenomes of soil samples have been sequenced to assess the population dynamics of *lin* genes in contaminated sites. These studies revealed that the prevalence of upstream *lin* genes is correlated with the level of HCH contamination in the soil, suggesting selective pressure drives acquisition of the transmissible *lin* genes in HCH contaminated soil (Sangwan *et al.* 2012).

Although most characterized strains have a full complement of *lin* genes, there is a growing body of evidence from newly sequenced strains that key components of the pathway frequently are missing (Dogra *et al.* 2004; Kohli *et al.* 2013; Kumar Singh *et al.* 2013; Mukherjee *et al.* 2013). Given the apparent nature of pathway acquisition through a series of horizontal transfer events, these missing genes are likely to reflect early stages in the acquisition of the pathway, with some species yet to acquire some genes. With an incomplete pathway, these strains are unlikely to be able to completely mineralize HCH, instead converting it cometabolically. Many HCH-degrading strains have been poorly characterized in this regard, however, leaving open the possibility that there may be alternate steps in HCH degradation yet to be discovered. An additional limitation with the currently sequenced species is that, apart from *S. japonicum* UT26, the others have all been isolated from Indian soil samples, with many being isolated from the same field. Any insights gained from these strains might therefore be specific to the particular environmental conditions they encounter and restrict our ability to draw broader conclusions about the evolution of the *lin* pathway.

Here, we present an analysis of three HCH-degrading strains (*Sphingobium czechense* LL01, *Novosphingobium barchaimii* LL02, and *Sphingobium baderi* LL03) isolated from soil samples at a Czech lindane-manufacturing site (Kaur *et al.* 2013; Niharika *et al.* 2013a,b). Strain LL01 has been reported to degrade α-, β-, γ-, and δ-HCH and LL03 to degrade only α-, γ-, and δ-HCH. Strain LL02 has been reported to degrade technical-HCH (a mix of HCH isomers); however, it is unknown which pure isomers it is capable of degrading. No information has yet been reported on how the HCH degradation in these strains compares with other, well-characterized, strains or on the relative rates of isomer degradation in each strain. Both of these features are known to be affected by the *lin* gene complement of a strain (Sharma *et al.* 2011; Wu *et al.* 2007)

We first compared the degradation patterns and metabolites of α-, β-, γ-, and δ-HCH in each strain with the well-characterized strain *S. japonicum* UT26, which degrades all four isomers. We were able to confirm the previously published abilities of LL01 and LL03 and show that *N. barchaimii* LL02 degrades α-, γ-, and δ-HCH but with a shift in isomer preference, degrading δ-HCH much faster and γ-HCH much slower than the other strains. We then sequenced the genomes of the three Czech strains to compare them with the published genomes of UT26 (isolated in Japan) and six other strains isolated in India. The comparative genomic analysis presented here finds partial *lin* pathways in half of the studied strains, with variable organization of those genes that are present. It also highlights the key role of IS*6100* elements in *lin* gene transmission, showing a variety of insertion sites for IS*6100* elements around the *lin* genes, and the first association of IS*6100* with the *linGHIJ* cluster. Finally, we identify a LinA variant in *N. barchaimii* LL02 that is presumed to be responsible for the shift in isomer preference.

## Methods

### Strains and media

Bacterial strains used in this study were obtained from the DSMZ (www.dsmz.de) under the accession numbers DSM-16413 (*S. japonicum* UT26), DSM-25410 (*S. czechense* LL01), DSM-25411 (*N. barchaimii* LL02), and DSM-25433 (*S. baderi* LL03). Quarter-strength Luria Bertani medium (QSLB; 2.5 g of tryptone, 1.25 g of yeast extract, 2.5 g of NaCl, 20 g of sodium succinate, and 20 mL of glycerol per liter) was used for the growth of these strains at 30°.

### Degradation assays

Seed cultures of each strain were prepared by inoculating glycerol stocks into QSLB and incubating at 30° overnight with shaking (180 rpm). The seed cultures were then centrifuged at 8000*g* for 10 min at 4° and the pellet resuspended in QSLB. After standardizing to an OD_600_ of 0.3, the cells were added (1% v/v) into a 500-mL flask with 20 mL of QSLB containing either α-, β-, γ-, or δ-HCH at a concentration of 0.5 ppm. Uninoculated QSLB containing HCH was used as a negative control, and all treatments were tested in triplicate cultures.

Samples of growth media from each flask (500 µL) were taken at 0, 0.5, 1, 4, 8, 24, 32, and 52 hr after inoculation. Because of the slow degradation of γ-HCH by strain LL02, an additional sample was taken at 96 hr after inoculation. Residual HCH and any metabolites were extracted with equal volumes of hexane and analyzed by gas chromatography using an Agilent 7890A gas chromatograph with an electron capture detector and a BPX-50 column (length: 30 m, internal diameter: 0.32 mm, film thickness: 0.25 µm). The temperature conditions for the analysis were held isothermally at 100° for 5 min, followed by an increase at a rate of 10° per minute to 200°, which was held for a further 5 min. Quantitation of HCH isomers was performed with standard curves produced from serial dilutions of the control flask media. A qualitative assessment of the metabolites produced by each strain was made by comparison to the retention time of 1,2,3-TCB, 1,2,4-TCB, and 1,3,5-TCB as authentic standards (Sigma-Aldrich) or to the pentachlorocyclohexane (PCCH) or pentachlorocyclohexanol metabolites produced with purified LinA and LinB respectively from the relevant HCH isomer (Trantirek *et al.* 2001).

### Genome sequencing and analysis

Genomic DNA for *S. czechense* LL01, *N. barchaimii* LL02 and *S. baderi* LL03 was prepared using the QIAGEN Genomic Tip kit (QIAGEN) following the manufacturer’s protocol. DNA was sequenced on an Illumina HiSeq2000 by Macrogen (Seoul, Korea). Both a short (500 bp) and long (5 kb) insert library was prepared and 100-bp paired reads were obtained from each sample. The initial reads were quality filtered (minimum average quality = 3) and trimmed (minimum quality = 20) using PrinSeq (Schmieder and Edwards 2011) and then downsampled to 100−120× coverage based on predicted genome size. FastQC was used to inspect the reads for quality issues before proceeding with the assembly.

Initial assemblies were constructed using the ABySS assembler (Simpson *et al.* 2009) with a range of k-mer lengths and the best assembly based on the contig N50 metric for each strain was selected for scaffolding using the matepair reads. Gaps in the final assembly were closed with GapFiller (Nadalin *et al.* 2012) and annotation of the final assemblies was performed using PGAAP [National Center for Biotechnology Information (NCBI)].

Concurrent with this work, the genome of *S. baderi* LL03 has been independently sequenced using the 454 GS FLX Titanium technology. Comparison with the assembly produced in this study from Illumina sequencing indicates that the two have near identical sequences, with 98.2% of the 454 assembly aligning to the Illumina assembly with greater than 99.8% identity. All analysis described has been carried out on the Illumina assembly. The Whole Genome Shotgun sequences for *S. czechense* LL01, *N. barchaimii* LL02, and *S. baderi* LL03 have been deposited at DDBJ/EMBL/GenBank under the accession numbers JACT00000000, JACU00000000, and JACV00000000 respectively. The versions described in this paper are JACT01000000, JACU01000000, and JACV01000000.

In addition to the aforementioned sequences, genome sequences for *S. japonicum* UT26 (GCF_000091125), *S. indicum* B90A (AJXQ01000000), *Sphingomonas* sp. MM-1 (GCF_000347675), *Sphingobium* sp. HDIP04 (ATDO01000000), *S. chinhatense* IP26 (AUDA01000000), *S. quisquiliarum* P25 (ATHO01000000), *S. ummariense* RL-3 (AUWY01000000), *Sphingobium* sp. SYK-6 (GCF_000283515), *Sphingomonas wittichii* RW-1 (GCF_000016765), and *Novosphingobium aromaticivorans* DSM12444 (GCF_000013325) and the *linB* containing plasmid pLB1 (NC_008330) were used in our analysis. Average nucleotide identities (ANIs) of all pairwise comparisons were calculated with JSpecies (Richter and Rosselló-Móra 2009). Whole-genome alignments were performed against UT26, LL01, LL02 and LL03 using MUMmer (Delcher *et al.* 2002) and regions of horizontal transfer in each target genome were predicted with the Genomic Island Suite of Tools (GIST), which combines the results of several common horizontal transfer prediction tools to produce a single, improved estimate (Hasan *et al.* 2012). This information was visualized using Circos (Krzywinski *et al.* 2009).

The *lin* genes of UT26 (*linA*; SJA_C1-18560, *linB*; SJA_C1-19590, *linC*; SJA_C1-00590, *linD*; SJA_P1-01390; *linE*; SJA_P1-01430, *linR*; SJA_P1-01440; *linF*; SJA_C2-04820, *linG*; SJA_C2-05210, *linH*; SJA_C2-05220, *linI*; SJA_C2-05200, *linJ*; SJA_C2-05190, *linK*; SJA_C1-00210, *linL*; SJA_C1-00220, *linM*; SJA_C1-00230, *linN*; SJA_C1-00240,) were used as queries for TBLASTX searches in the newly sequenced strains. The top hits of each search were then aligned to the non-redundant NCBI database to confirm their assignment. The genomic regions containing *lin* genes were aligned pairwise with MEGABLAST and displayed with genoPlotR (Guy *et al.* 2010) to examine the conserved regions between the studied sequences.

## Results

### Degradation of HCH

**Figure 1:**
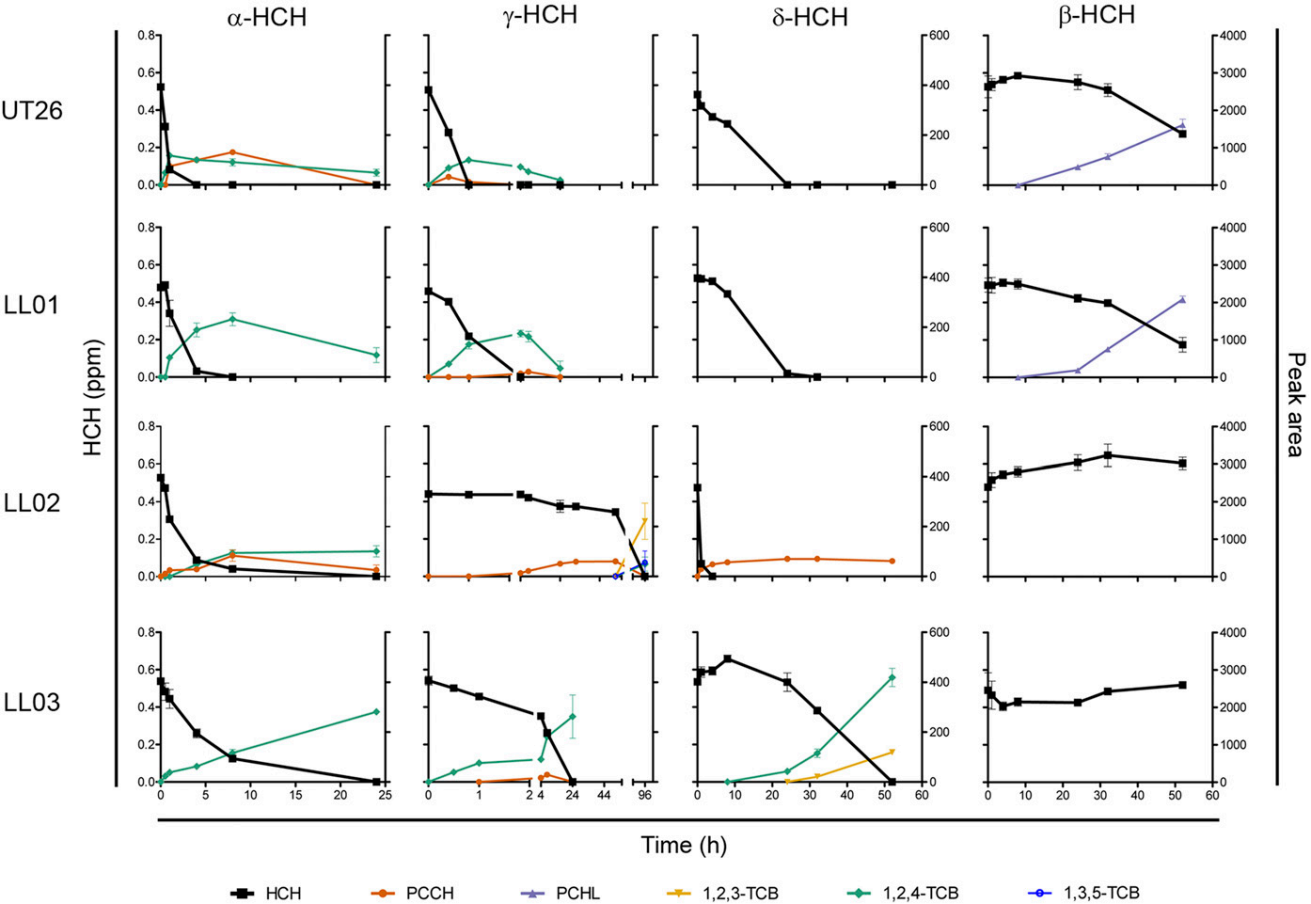
Degradation assays for HCH isomers. Degradation of α-HCH, β-HCH, γ-HCH, and δ-HCH (left-hand axis) by UT26, LL01, LL02, and LL03 and relative quantification of metabolites by peak area (right-hand axis). Time postinoculation is shown on the bottom axis and the timescale for the γ-HCH assay has been adjusted to observe both the very fast (less than 1 hr) and very slow (96 hr) degradation of γ-HCH by different strains. Values are the mean of three biological replicates, with standard deviations. Identity of metabolites was confirmed by comparison to authentic standards or to metabolites produced with purified UT26 LinA or LinB enzymes. HCH, hexachlorocyclohexane; PCCH, pentachlorocyclohexene; PCHL, pentachlorocyclohexanol; TCB, trichlorobenzene.

Growth assays with four HCH isomers allowed us to compare the abilities of LL01, LL02, and LL03 to degrade the various HCH isomers with those of the well characterized strain UT26 (Figure 1). We were able to confirm the ability of LL01 to degrade all four isomers tested (relative rates: α = γ > δ >> β), and of LL03 to degrade α-, γ-, and δ-HCH (α > γ >> δ) (Kaur *et al.* 2013; Niharika *et al.* 2013a). LL02 was found to degrade only α-, γ-, and δ-HCH (δ > α >> γ). Compared with UT26, LL01 exhibited a similar degradation pattern for each isomer, whereas LL03 was much slower than UT26 for the three isomers it could degrade. LL02 had a novel degradation pattern, with slower γ-HCH and faster δ-HCH degradation than the other strains. Although LinA variants are known to affect the α-HCH enantiomer specificity or the α/γ isomer preference (Sharma *et al.* 2011; Suar *et al.* 2005), this is the first strain reported to degrade δ-HCH faster than all other isomers.

Metabolites produced in the growth assays were identified by comparison of their retention time to known standards. Strain LL01 produced the same metabolite profile for each isomer as UT26, with degradation of β-HCH−producing pentachlorocyclohexanol and the remaining isomers producing pentachlorocyclohexane (PCCH), and trichlorobenzene (TCB). β-PCCH was not directly observed in the degradation of α-HCH by strain LL01 but is inferred from the observed TCB metabolite. As the bulk of the δ-HCH degradation occurred between the 8- and 24-hr samples, no metabolites for δ-HCH degradation were observed in UT26 and LL01.

Strain LL03 produced the same metabolites as UT26 for α-HCH and γ-HCH but produced 1,2,4-trichlorobenzene (1,2,4-TCB) and 1,2,3-trichlorobenzene (1,2,3-TCB) when incubated with δ-HCH and could not degrade β-HCH at all. The δ-HCH metabolites produced by LL03 have been observed when δ-HCH is incubated with purified UT26 LinA enzyme but not in cell cultures of various strains, as δ-HCH is preferentially degraded by LinB (Geueke *et al.* 2013). These metabolites, combined with the lack of degradation of β-HCH (a LinB-initiated degradation), are consistent with the claim that LL03 lacks a *linB* gene, leading to LinA-initiated degradation pathways (Kaur *et al.* 2013).

Strain LL02 is similar to LL03 in that degradation of α-, γ-, and δ-HCH all appears to be LinA-initiated, producing their respective PCCHs, and no degradation of β-HCH is observed. The largest difference between LL02 and LL03 comes in the further degradation of γ-PCCH, with LL03 (and UT26) accumulating 1,2,4-TCB as a dead end product, whereas LL02 accumulates 1,2,3-TCB, 1,2,4-TCB, and 1,3,5-TCB. This is the first report of the 1,2,3- and 1,3,5-TCB isomers being observed from γ-HCH degradation. It is unclear why different TCB isomers would be produced in this degradation, as 1,2,4-TCB is proposed to be spontaneously produced from tetrachlorocyclohexadiene, the putative final product of LinA activity (Nagata *et al.* 1993b). However, as 1,2,4-TCB is identified in degradation assays with purified LinA enzyme, the altered metabolite profile provides additional evidence for the presence of a novel LinA variant in strain LL02.

### Genome sequences of Czech strains

For each strain, short and long insert libraries were prepared and sequenced using Illumina HiSequation 2000. After quality control, the short insert sequences were assembled using the ABySS assembler and the best assembly for each strain was selected for scaffolding and gap filling using the matepair reads. The final assemblies produced draft genomes comprising 21, 26, and 82 contigs for LL01, LL02, and LL03, respectively (Table 1). These contigs were assembled into 18, 19, and 11 scaffolds, respectively. The LL01 assembly contains four chromosomal scaffolds (totaling 4.1 Mb in size) and a plasmid scaffold of 186 kb. The LL02 assembly contains 10 chromosomal scaffolds and a plasmid scaffold of 242 kb. In the LL03 assembly, 98% of the genome is contained in four circular scaffolds, comprising two chromosomal scaffolds (3.4 Mb and 641 kb) and two plasmid scaffolds (510 kb and 34 kb). The total genome size and GC% content for each assembly is consistent with previously sequenced genomes from the relevant genus (Copley *et al.* 2012; Dong *et al.* 2014; Gan *et al.* 2012; Luo *et al.* 2012; Masai *et al.* 2012). Annotation of each genome was performed using the PGAAP (Angiuoli *et al.* 2008).

**Table 1:** Assembly summary: summary of the final genome assemblies produced for this study

Strain	Number of Contigs	Contig N50	Number of Scaffolds	Final Assembly Size, Mbp	G/C Content, %	Chromosomal Scaffolds, (Total Size (Mbp)	Plasmid Scaffolds, Total Size (kbp)
LL01	21	480	18	4.6	63.6	4 (4.1)	1 (186)
LL02	26	797	19	5.3	64.0	10 (4.9)	1 (242)
LL03	82	127	11	4.7	63.5	2 (4)	2 (544)

### *lin* genes and IS*6100* elements in LL01, LL02, and LL03

We identified the *lin* genes present in LL01, LL02, and LL03 by homology to the protein sequences from UT26 using the top BLAST hits (E-value < 1e-20) (Table S1). Each potential hit was then used as a BLAST query against the NCBI nr database to confirm that its closest match was a *lin* gene. There is consistent representation of the conserved downstream elements of the pathway representing core sphingonomad functional systems, with each strain containing a *linKLMN* cluster encoding an ABC transporter and most components of the *linGHIJ* cluster encoding the β-ketoadipate pathway (Nagata *et al.* 2010). In strain LL01, however, the *linGHIJ* cluster is disrupted by an IS*6100* element, causing the transcriptional regulator *linI* to be interrupted and *linJ* (encoding an acetyl-CoA acetyltransferase) to be lost.

Unlike the core functional genes, there is considerable variation in the presence of the remaining *lin* genes (*linA-F*) in the three strains. In LL01, we identified a complete upstream pathway, with *linA*, *linB* (two copies) and *linC* all present; however, *linRED* and *linF* are missing. In LL02 we only identified *linA* and an IS*6100*-truncated *linF*, whereas in LL03 we only identified *linA*, *linF*, and a disrupted *linR*. These variable genes (*linA-F*) comprise the HCH-specific component of the pathway, in contrast to the more general role performed by the more consistently represented core functional genes. The presence or absence of *linA* and *linB* in each of the three strains is sufficient in these cases to explain which HCH isomers each strain could degrade. Further sequencing of additional isolates would identify the extent of upstream *lin* gene variation and its association with HCH isomer degradation.

**Figure 2:**
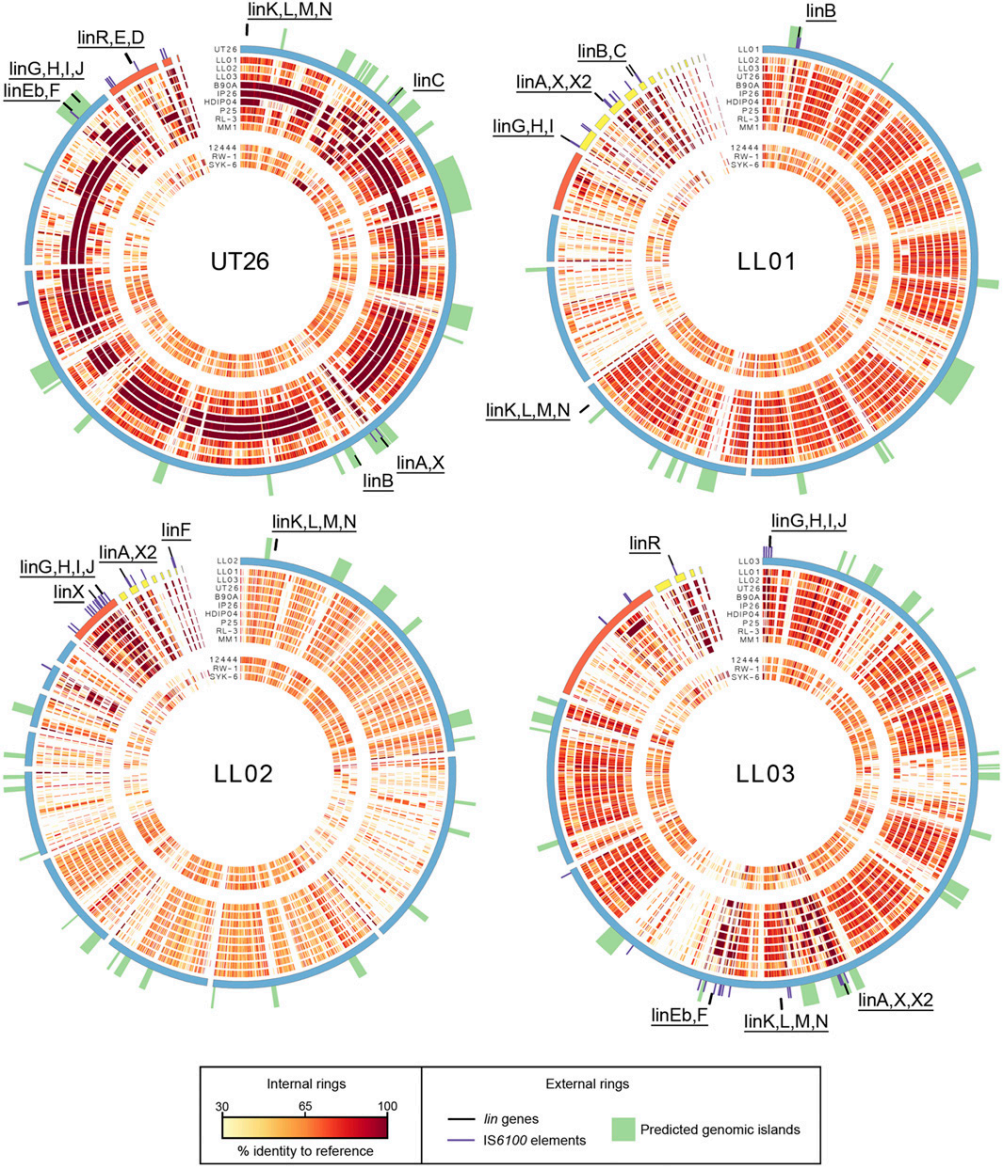
Whole-genome alignments of sequenced strains. Whole-genome alignments of the other 12 genomes (see the *Materials and Methods* section for names and accession numbers) to each of the UT26, LL01, LL02, and LL03 genomes. The color gradient of the inner rings indicates homology to the reference genome sequence. On display starting from the innermost ring are three non−HCH-degrading strains, then 10 HCH-degrading strains, with the reference genome the outermost ring. Reference chromosomes or scaffolds predicted to be from chromosomes are colored blue, plasmids are colored red, and small scaffolds that cannot be unambiguously determined to be from either chromosomal or plasmid origin are colored yellow. The positions of IS*6100* elements (purple) and *lin* genes (black) in the reference genome are indicated. Predicted genomic islands are highlighted in green. HCH, hexachlorocyclohexane.

We also assessed each genome for the prevalence of IS*6100* elements, given their known association with the *lin* genes (Dogra *et al.* 2004). We identified 14, 19, and 24 IS*6100* elements in LL01, LL02, and LL03, respectively, either confirmed through a match to the IS*6100* transposase (accession BAI98840) or inferred by a match to two 15 bp IS*6100* inverted repeats (GGCTCTGTTGCAAA) across contig boundaries (Mahillon and Chandler 1998). These numbers fall within the range seen among sequenced HCH-degrading strains (13−26) and are much greater than seen in the three non−HCH-degrading strains from the same genera used in our comparative analysis; *Novosphingobium aromaticivorans* DSM12444 (0), *Sphingomonas wittichii* RW-1 (1), and *Sphingobium* sp. SYK-6 (0) (Table 2). IS*6100* is not a *lin*-gene−specific transposable element, having also been associated with catabolic genes for nylon (*Flavobacterium* sp. KI72, *Pseudomonas* sp. NK87), carbazole (*Sphingomonas* sp. XLDN2-5), and nonyl-phenols (*Sphingomonas* sp. NP5), among others, however, only small numbers of IS*6100* elements (2−5) have been identified in these cases (Gai *et al.* 2010; Kato *et al.* 1994; Takeo *et al.* 2012; Yasuhira *et al.* 2007). The IS*6100* elements in LL01, LL02, and LL03 appear to be highly clustered around the *lin* genes, supporting the proposed involvement of IS*6100* in *lin* gene recruitment. In LL03 for example, the 19 IS6100 elements found on the 3.4 Mb main chromosome scaffold lie at a median distance of 25.5 kb from the nearest *lin* gene, with five found near *linGHIJ*, four near *linX* and *linA*, two near *linKLMN*, and seven near *linF* (Figure 2).

**Table 2:** IS*6100* elements in the studied genomes

	Confirmed	Putative	Total
LL01	12	2	14
LL02	17	2	19
LL03	24	0	24
B90A	0	20	20
HDIP04	4	9	13
RL-3	16	7	26
P25	23	2	25
IP26	16	10	26
UT26	14	NA*^a^*	14
MM-1	14	NA*^a^*	14
RW-1	1	NA*^a^*	1
SYK-6	0	NA*^a^*	0
DSM12444	0	NA*^a^*	0

Number of confirmed and predicted IS*6100* elements in the 13 genomes studied in this article. Confirmed elements were identified by a match to the IS*6100* transposase and putative elements were predicted from the presence of two IS*6100* terminal inverted repeat sequences across contig boundaries. In completed genomes, the inverted repeat sequence is only found associated with IS*6100* elements. NA, not available.

a Complete genome, no contig boundaries to assess putative IS*6100* elements.

### Comparison of Czech strains to other HCH-degrading strains

Previous genomic comparisons between UT26 and B90A have suggested that these two strains may have been derived from a common ancestor containing most of the *lin* genes (Sangwan *et al.* 2014). However, a similar analysis between UT26 and three other HCH-degrading strains (*Sphingobium* sp. TKS, *Sphingobium* sp. MI1205 and *Sphingomonas* sp. MM-1) suggested the independent acquisition of HCH degradation in each of the four strains (Nagata *et al.* 2014). The expanding number of genomes available for HCH-degrading strains allows us to test the relative contribution of these two modes of evolution more broadly in *lin* pathway evolution.

Whole-genome alignments of nine HCH-degrading strains (*S. czechense* LL01, *N. barchaimii* LL02, *S*. *baderi* LL03, *S. indicum* B90A, *S. chinhatense* IP26, *Sphingobium* sp. HDIP04, *S. quisquiliarum* P25, *S. ummariense* RL-3, and *Sphingomonas* sp. MM-1), and the three non−HCH- degrading strains mentioned previously, to UT26 reveal both modes of evolution noted previously (Figure 2). Three of the other HCH degraders, B90A, IP26, and HDIP04, all have very high homology (ANI of 97.1–97.7%) to large regions of the UT26 genome, indicating a recent common ancestor from which they may have obtained most *lin* genes. However, the remaining six strains, including all three Czech strains, show smaller regions of homology, at lower levels of identity to UT26 (ANI 75.3–81.9%), indicating more divergence among the shared set of genes common to all sphingomonads. Compared with this low overall level of homology, the high identity (generally >97%) of *lin* genes in these strains to UT26 indicates that they have likely been acquired horizontally, rather than through shared ancestry with UT26. The sequence composition of the genomic regions containing *lin* genes also suggests the acquisition by horizontal transfer; the GIST (Hasan *et al.* 2012) identified predicted genomic islands which contain all *lin* genes in UT26 except *linKLMN*.

The aforementioned alignments were repeated with each of LL01, LL02, and LL03 as a reference genome to determine whether these strains acquired their *lin* genes independently of each other (Figure 2). Unlike what was observed in the comparisons of UT26, these three showed no close relationship to any of the other genomes studied (LL01 ANI: 73.6–79.9%, LL02 ANI: 73.0–75.1%, LL03 ANI: 75.7–81.8%). Where small areas of strong homology exist between the strains, they are associated with the *lin* genes, IS*6100* elements or genomic islands predicted by GIST. This suggests that the distinct *lin* gene complements of each of these strains may reflect independent horizontal gene transfer events. This whole-genome analysis is consistent with phylogenies based on 16S sequences that place LL01, LL02, and LL03 relatively distant from other HCH-degrading strains (Kaur *et al.* 2013; Niharika *et al.* 2013a; Niharika *et al.* 2013b).

**Figure 3:**
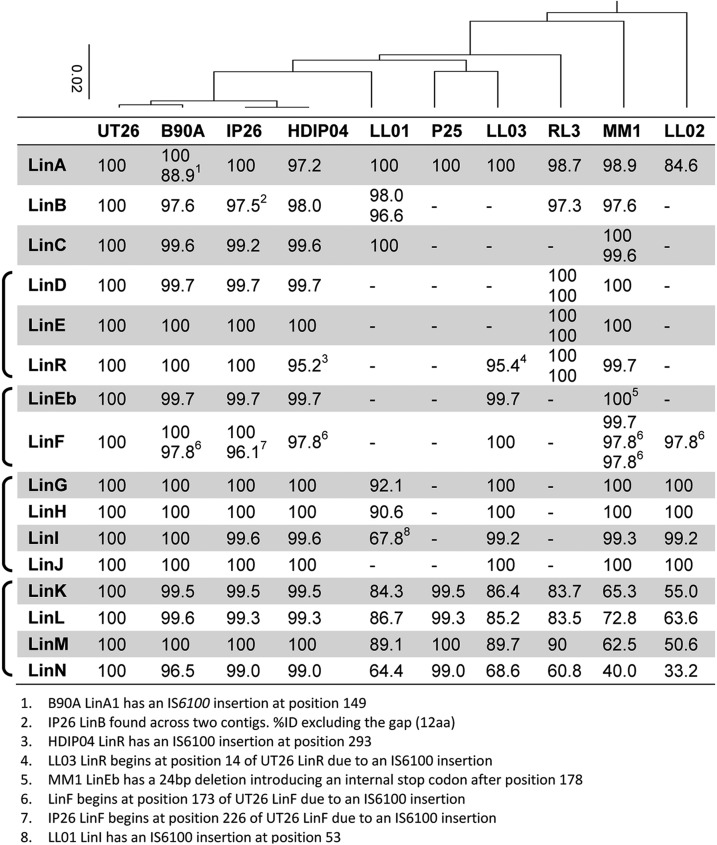
*lin* pathway composition of studied strains. Presence of *lin* genes in the 10 HCH-degrading strains studied, along with the percent identity of each encoded protein to the equivalent in UT26. Genes grouped in operons are indicated with braces to the left of the gene names. Multiple values indicate duplicate copies of the gene in a strain. The neighbor-joining phylogeny above the strain names was produced from a clustalw alignment of the 16S rDNA sequences (UT26: AF039168, B90A: AY519129, IP26: EF190507, HDIP04: EF424393, LL01: JN646865, P25: EU781657, LL03: JN695620, RL-3: EF207155, MM-1: CP004036;G432_r19183, LL02: JN695619) of each strain with *E. coli* (ECK3843) as an outgroup (not shown). HCH, hexachlorocyclohexane.

As indicated previously, there is considerable variation in *lin* gene complement among the 10 HCH-degrading genomes considered here (Figure 3). The four very closely related strains (UT26, B90A, IP26, and HDIP04) each have a complete *lin* pathway, suggesting that it was acquired from their common ancestor. Of the remaining strains, only MM-1 has a complete pathway, whereas LL01, LL02, LL03, RL-3, and P25 have missing components. The selective pressure to acquire a *lin* pathway in HCH-contaminated soil suggests that these strains with partial *lin* pathways are likely in the early stages of pathway assembly, having acquired only a few of the required genes, but we cannot discount the possibility that some represent instances of gene loss (Sangwan *et al.* 2012).

There are also instances of multiple copies of the *linA-F* genes, with two copies of *linA* in B90A, two of *linB* in LL01, two of *linC* in MM1, two of *linRED* in RL-3, two of *linF* in B90A and IP26, and three of *linF* in MM1. In the case of *linF*, however, all extra copies are disrupted by IS*6100* elements, and it is unknown if they are functional. Multiple copies of the upstream genes may relax selective constraints, allowing functional specialization of the duplicated copies as seen in the enantioselective degradation of α-HCH by LinA1/LinA2 in B90A (Suar *et al.* 2005), or it may increase the expression of the gene, with *linA*, *linB*, and *linC* all being expressed constitutively (Suar *et al.* 2004). Little variation is seen in protein sequences for these genes however, with almost all copies observed having very high identities to the UT26 sequences (Figure 3). This paucity of variation is observed in all *lin* genes except *linKLMN*, further supporting the prediction that these genes, including core downstream components such as the β-ketoadipate pathway encoding *linGHIJ*, have been acquired by horizontal transfer. The greater variation among *linKLMN* sequences suggests that this cluster has not undergone horizontal transfer, but has been acquired from a common ancestor.

### Genomic organization of *lin* genes in HCH-degrading strains

**Figure 4:**
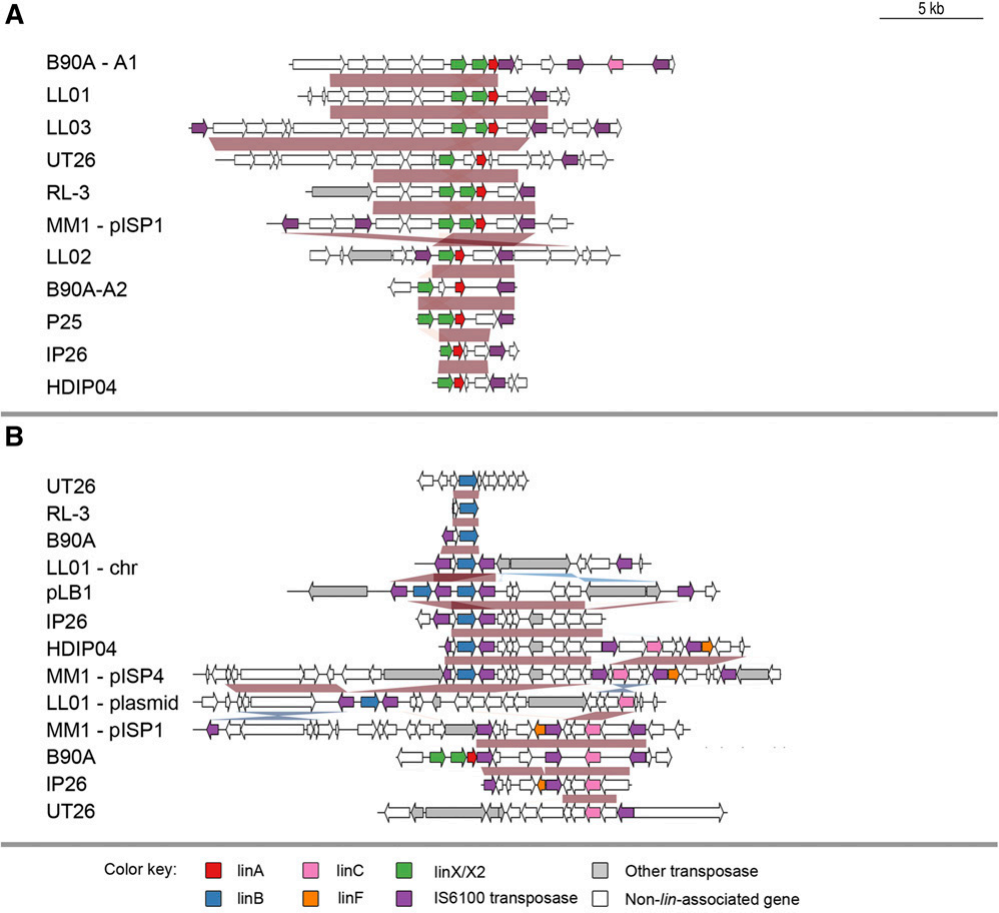
Genomic organization of upstream *lin* genes. Alignments detailing strong conservation and IS*6100* involvement immediately surrounding the upstream *lin* genes in HCH-degrading strains. Alignments of the regions surrounding (A) *linA* and (B) *linB* and *linC* were performed with MEGABLAST and ordered to give maximal pairwise alignment lengths. Conserved regions are indicated by red shading where the matches are in the same orientation and by blue shading where the matches are in reverse orientation. Also found associated with these genes are an IS*6100* truncated *linF* and *linX/linX2* (*linC*-like sequences not necessary for HCH degradation). HCH, hexachlorocyclohexane.

**Figure 5:**
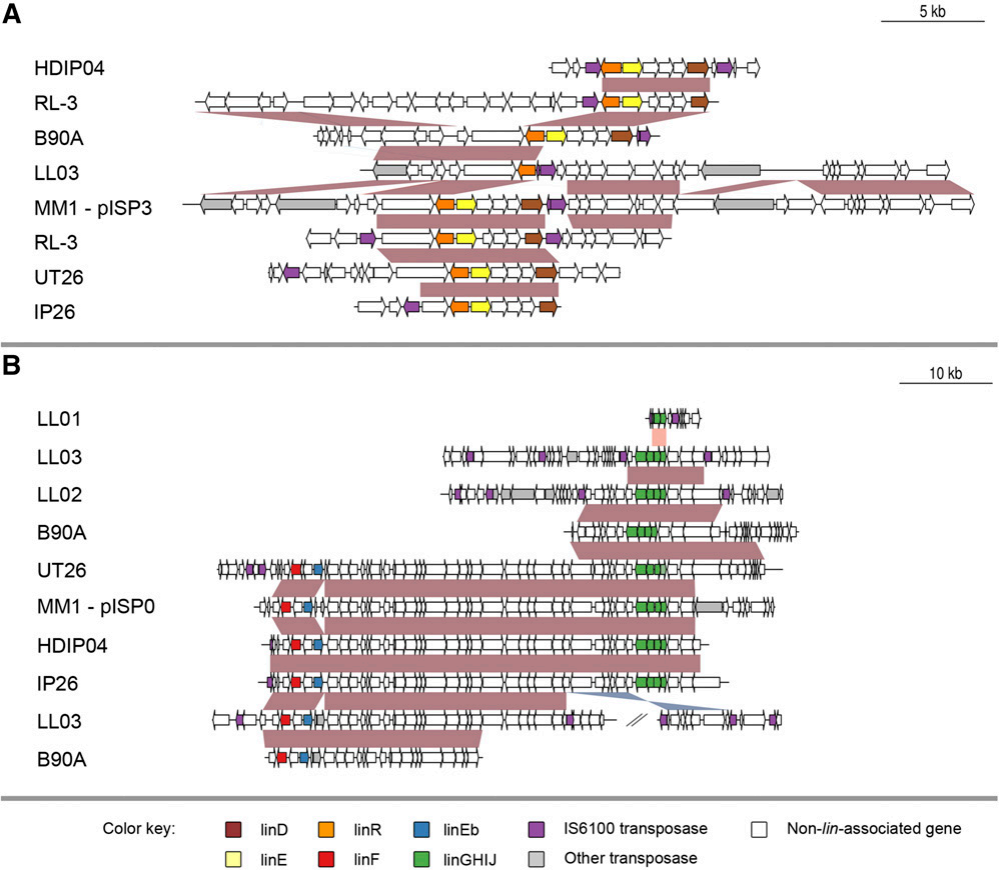
Genomic organization of downstream *lin* genes. Alignments detailing conservation and rearrangements in the genomic regions of the downstream *lin* genes. Alignments of the regions surrounding (A) *linDER* and (B) *linFEb* and *linGHIJ*, were performed with MEGABLAST and ordered to give maximal pairwise alignment lengths. Conserved regions are indicated by red shading where the matches are in the same orientation and by blue shading where the matches are in reverse orientation. Note that association of IS*6100* elements with *linGHIJ* is unique to strains LL01, LL02 and LL03.

We looked at microsynteny at the *lin* gene loci to investigate the extent of the horizontally transferred regions and their ongoing rearrangement (Figure 4 and Figure 5). Alignments of genomic regions from the above ten genome sequences plus a *linB* containing plasmid isolated exogenously from HCH-contaminated soil (Miyazaki *et al.* 2006) revealed high levels of conservation around the *lin* genes, generally delimited by IS*6100* elements. These regions range from many kilobases in length in the case of the downstream *lin* genes to just a few hundred base pairs for some of the upstream *lin* genes. Because longer horizontally transferred sequences are more likely to have a higher fitness cost, the size of these conserved regions may be related to the extent of horizontal transfer of each region. The high level of conservation of gene content and intergenic sequence around all *lin* genes except *linKLMN* (Supporting Information, Figure S2) suggests that each has been derived from a single original source.

One major difference between strains is the location of IS*6100* insertions around certain *lin* gene regions, in particular *linA* and *linRED*. In the case of *linA*, we identified five different insertion sites downstream of *linA*. The UT26 sequence has an insertion 3638 bp downstream of *linA*, while there are also insertions at 1550 bp (LL01, LL02, LL03, RL-3, MM1, B90A-A2, and P25), 1254 bp (IP26), and 1197 bp (HDIP04) downstream of *linA*, as well as the B90A_LinA1_ insertion 444 bp into the *linA* gene (Figure 4A). Of the eight *linRED* clusters identified, five have an IS*6100* element upstream of the cluster (UT26, IP26, HDIP04, and two clusters in RL-3). Each of these IS elements is located in a different position (Figure 4C). These examples show the continuing activity of IS*6100* in HCH-degrading strains and their potential for ongoing involvement in the transfer and evolution of the *lin* pathway.

Not all *lin* genes show such variability, however. The location of IS*6100* around *linB* is quite consistent in all strains, with insertion sites 307 bp upstream and 105 bp downstream of the gene (Figure 4B). The only exceptions to this are found in IP26, where the insertion site is 387 bp upstream, and in UT26, which lacks any flanking IS elements around *linB*. As will be discussed below, this lack of variability in IS*6100* arrangement around *linB* may be because it is a recent acquisition that has been selectively favored to increase *linB* transmissibility.

### *N. barchaimii* LL02 contains an uncharacterized LinA variant

**Figure 6:**
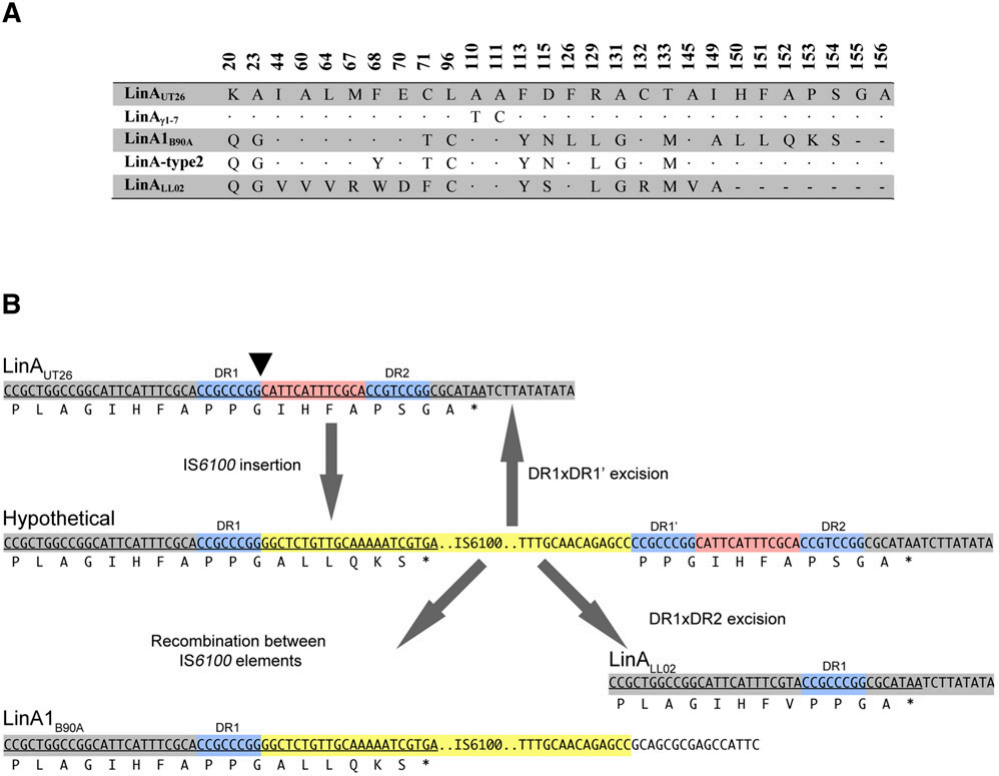
Comparison of LinA sequence variants. (A) Amino acid differences between major LinA variants and the uncharacterized LinA of LL02. (B) Proposed mechanism for the IS6100-mediated 21-bp deletion in LL02. The initial insertion site of the IS*6100* cointegrates (yellow) in a LinA_UT26_-like sequence is marked with a black triangle. The insertion induces the duplication of 8bp at the insertion site (DR1 and DR1´ in blue). IS*6100* elements are capable of reversion through homologous recombination between the induced direct repeats (DR1×DR1´). A near-identical sequence (DR2) is slightly further downstream of the insertion, however, and recombination between DR1 and DR2 gives rise to the sequence observed in LL02, with the deletion of one DR and the intervening sequence (colored red). Also shown is the sequence of LinA1 in B90A, containing the IS*6100* insertion and a different downstream sequence. This has likely arisen through homologous recombination between IS*6100* elements, deleting the intervening region containing the original C-terminus of LinA.

Closer analysis of the *linA* gene sequence in *N. barchaimii* revealed that it encodes an uncharacterized LinA enzyme (Figure 6A). Compared with the two B90A variants LinA1 (accession number AAN64239) and LinA2 (ACM48253), the LinA_LL02_ contains 11 and 17 amino acid differences respectively, as well as a seven amino acid deletion at the C-terminal end. Many of the mutations in LinA_LL02_ are clustered near the catalytic histidine (H73) and affect amino acids predicted to form the active site (Okai *et al.* 2010). In other characterized LinA enzymes, small changes to the amino acid sequence are capable of altering the α-HCH enantiomer preference or the α-/γ-HCH isomer preference. The mutations in LinA_LL02_, therefore, provide candidates for the shift in isomer preference that sees strain LL02 preferentially degrading δ-HCH. Further work characterizing LinA_LL02_ would confirm this hypothesis and identify which mutations are necessary for the altered phenotype. A sequence identical to LinA_LL02_ has also been recently identified in a metagenomic screen of Indian soil samples, indicating that it is not a geographically restricted variant (Lal *et al.* 2013).

## Discussion

### Recruitment of *lin* genes in sequenced genomes

Until recently, almost all characterized HCH-degrading strains contained a full complement of *lin* genes [*Sphingomonas* sp. γ12-7, the only exception, being deficient in *linRED* (Böltner *et al.* 2005)]. However, emerging evidence suggests that there is considerable variability in the recruitment of the pathway, with eight of the ten genomes studied here either missing or containing multiple copies of the *linA-F* genes. Of these variable genes, only *linA* is present in all strains, suggesting that it is necessary for a strain to be isolated in an enrichment screen for HCH degradation. *linA* also appears to be sufficient for isolation in an enrichment screen, as it is the only upstream *lin* gene found in *S. quisquiliarum* P25. The remaining genes (*linB-F*) are each absent in several strains and, given the horizontal transfer involved in pathway acquisition, likely represent early stages of pathway evolution, having acquired some *lin* genes but not all. These strains may therefore provide a valuable resource for studying the gradual accumulation of the *lin* genes and what function individual elements may serve in the absence of a full pathway.

We cannot be certain that these strains are yet to acquire some *lin* genes, however, because they are known to be highly mobile as the result of IS*6100*-mediated genome rearrangements (Nagata *et al.* 2011). Laboratory studies have shown that *lin* genes are lost rapidly when the strain is no longer exposed to HCH, with *Pseudomonas aeruginosa* ITRC-5 losing *linA-E* when grown in rich media (Singh *et al.* 2007). Some of the cases of missing *lin* genes may therefore be due to gene loss events, rather than the strain not yet acquiring the gene. This is the most likely explanation for some genes in LL03, as an IS*6100*-interrupted *linR* suggests that an IS*6100*-mediated rearrangement is the cause of *linED* missing from the genome. The situation in the Czech strains is further complicated because preliminary data suggested that they were able to grow on HCH as a sole carbon source (Kaur *et al.* 2013; Niharika *et al.* 2013a,b); however, we have been unable to replicate this phenotype. This finding suggests that these strains may have undergone secondary loss of some *lin* genes, during either preservation at the stock center or in the process of resuscitation, making it impossible to determine the original *lin* complement of these lines. The analysis of isolated cultures may therefore provide an incomplete picture of *lin* pathway evolution and need to be interpreted in light of other approaches, such as metagenome sequencing, that look at pathway organization in soil populations.

Where instances of missing *lin* genes can be confirmed in natural populations, there are two main proposals for how these strains are able to degrade HCH. It is possible that the functions performed by missing *lin* genes may be carried out by other unknown enzymes in the organism. As an alternative, the complementing functions may be carried out by other members of the microbial community. Both of these hypotheses have been supported in different strains. *Xanthomonas* sp. ICH12 provides an example of the former, with the identification of a novel metabolite after PCCH production (Manickam *et al.* 2007). The latter hypothesis is supported by studies into microbial consortia isolated from HCH-contaminated Indian soil samples, in which the community was able to degrade HCH far more effectively than any of the individually isolated elements (Elcey and Kunhi 2010; Manonmani *et al.* 2000).

Also informative in respect of the *lin* pathway’s origins are the genomic regions immediately surrounding the *lin* genes. In almost all cases, the genomic context of each *lin* gene (including flanking genes and intergenic regions) is highly conserved (*e.g.*, 100% match for the 1 kb downstream of *linA*). The conserved region generally extends out to a mobile genetic element, usually an IS*6100* element, and then abruptly ceases. Together with the whole-genome analysis suggesting multiple, independent acquisitions of the *lin* genes, these data suggest that each *lin* gene has a single evolutionary origin and then is rapidly distributed across species through horizontal transfer events.

*linB* is an interesting case because there appear to be two potential mechanisms of transfer. In UT26, *linB* is situated chromosomally in a GIST-predicted genomic island (Figure 2). Closer inspection of the predicted region identified it as a putative 73-kb integrative and conjugative element (ICE). ICEs (also known as conjugative transposons) are widely dispersed, horizontally transferred elements that encode their own conjugative transfer and integration/excision from a host genome (Guglielmini *et al.* 2011; Wozniak and Waldor 2010). This 73-kb region on the UT26 first chromosome contains the integrase, relaxase, and recombination directionality factor required for excision and integration of the region (Ramsay *et al.* 2006), as well as a type IV secretory system that conjugatively transfers the excised element between cells. These elements are known to carry accessory genes responsible for a range of functions including pathogenicity (Chuzeville *et al.* 2012; Seth-Smith *et al.* 2012), symbiosis (Sullivan *et al.* 1995; Sullivan *et al.* 2002), antibiotic resistance (Michael *et al.* 2012; Rodríguez-Blanco *et al.* 2012), and xenobiotic catabolism (Gaillard *et al.* 2006; Shettigar *et al.* 2012). In all other sequenced genomes containing a *linB*, however, the gene is flanked by two IS*6100* elements, with intergenic sequence 307 bp upstream and 105 bp downstream identical to the UT26 sequence (Figure 4B). As IS*6100* elements only very rarely excise themselves without leaving a copy of the element, it is highly likely that the IS*6100-linB*-IS*6100* element is derived from the *linB* containing ICE seen in UT26. Thus, Although *linB* may initially have been acquired by conjugation and integration of an ICE, it appears that the acquisition of flanking IS*6100* elements has subsequently been key to the widespread prevalence of *linB* in isolated strains. With high HCH concentrations appearing to impart a selective pressure for acquisition of the *lin* genes (Sangwan *et al.* 2012), an increase in IS*6100* activity could provide a possible mechanism.

### IS*6100* involvement in *lin* gene acquisition

The involvement of IS*6100* in *lin* gene acquisition is still clearly an active process, with a variety of IS*6100* element insertion sites identified. That these elements tended to define the limits of conserved regions around the *lin* genes is further evidence for the importance of IS*6100* in their horizontal transfer, as noted above. In addition to the widespread association of IS*6100* with the upstream *lin* genes, the new genomes sequenced for this study reveal the association of IS*6100* elements with the genes of the β-ketoadipate pathway (*linGHIJ*). This is the first time that IS*6100* elements have been found to be associated with these downstream components of the HCH degradation pathway. The fact that this arrangement is found in all three Czech strains suggests that, like the aforementioned *linB*, the acquisition of flanking IS*6100* elements may be the key to widespread horizontal transfer.

The clustered arrangement of IS*6100* with so many of the *lin* genes adds further evidence for a selectively favored increase in IS*6100* activity and *lin* gene transmissibility. In HCH-contaminated soil, an increase in IS*6100* activity would introduce a number of new elements in the genome. Any new IS*6100* elements inserted near *lin* genes will be favored in due course as they increase the chance of *lin* genes being passed on to other community members. A pressure to acquire the upstream *lin* genes in HCH contaminated soil is therefore likely to explain the newly identified association with the downstream genes.

**Figure 7:**
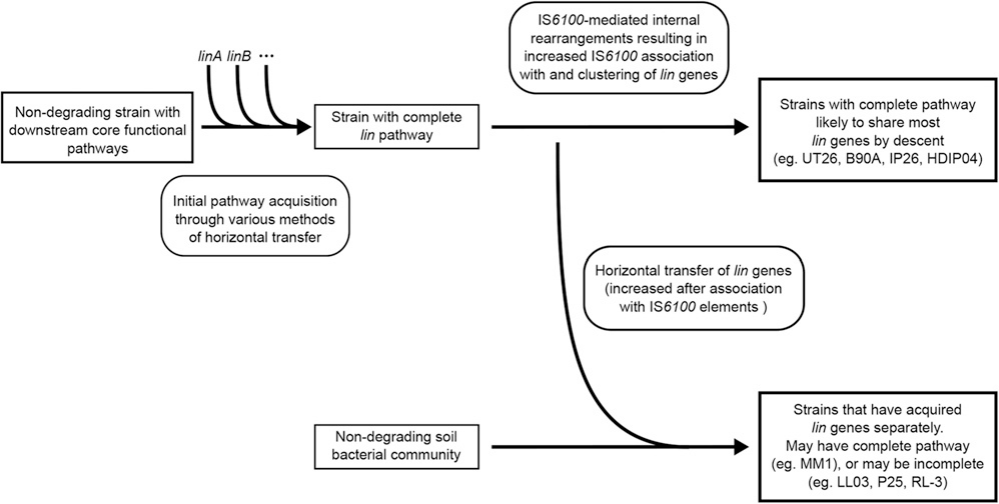
Hypothesized model of the *lin* pathway evolution. Proposed model of the processes involved in *lin* pathway evolution. Note that these steps are likely to be overlapping to some degree.

The increasing involvement of IS*6100* with the *lin* genes may indicate the beginning of a second phase in the evolution of the pathway (Figure 7). The first phase of evolution, the initial formation of the pathway, is proposed to have occurred with the recruitment of the upstream genes (through various means) into an ancestral strain containing the downstream genes that make up core sphingomonad functions (Nagata *et al.* 2014). The current phase appears to involve a consolidation of the pathway, in which we see an increase in IS*6100* activity that expands IS*6100* association to genes that were initially transferred through other means (*e.g.*, *linB*) and to downstream genes that were not previously known to be transferrable (*linGHIJ*). Although this appears to introduce more instability in the pathway than previously seen, it also increases the probability of the pathway being acquired after exposure to HCH. Indeed, the entire catalytic component of the *lin* pathway is now capable of IS*6100*-mediated transfer, opening up the possibility of transfer to species that lack the downstream components common to all sphingomonads.

### IS6100-mediated C-terminal truncation of LinA_LL02_

In addition to influencing the pathway organization, transposition and recombination of IS6100 elements can affect the sequence of Lin enzymes. In the case of LinA, an IS6100 insertion is seen at nucleotide position 444 of LinA1 found in B90A, which alters the C-terminal sequence of the protein. In LinA_LL02_, the seven amino acid C-terminal truncation is likely caused by imperfect excision of this IS6100 element (Figure 6B). Insertion of an IS6100 cointegrate is accompanied by an 8-bp direct repeat duplication of the insertion site (Herron *et al.* 1999). Homologous recombination can occur at this direct repeat, allowing reversion of the IS element insertion, although this occurs at very low rates (Weaden and Dyson 1998). Because this insertion in LinA occurs in a repeated GIHFAP motif, there is a nearly identical copy of the landing site direct repeat just downstream of the insertion (Figure 6B). An imperfect excision of the IS6100 element at this secondary direct repeat would give rise to the sequence observed in the genome of LL02.

The C-terminal extension is involved in stabilizing the LinA trimer and in closing off the active site once the substrate is bound (Macwan *et al.* 2013). The length of this extension is important for enzyme function, with two GIHFAP motifs producing enzymes with greater activity than those with one or three (Macwan *et al.* 2013). In the case of LinA_LL02_, the predicted loss of activity due to the single repeat may be compensated for by the C132R substitution. This substitution was identified by an *in vitro* mutagenesis assay as being responsible for a sixfold increase in LinA expression and solubility in *Escherichia coli* cells (Mencía *et al.* 2006). LinA_LL02_ is the first natural variant to be identified with this mutation.

### Continuing pathway evolution

Many different models have been proposed for the evolution of new metabolic pathways (Fondi *et al.* 2009). The *lin* pathway provides an excellent model to study the early evolution of new pathways, given how recently it has arisen. Our analysis of 10 sequenced genomes suggests that the *lin* pathway is following the piecewise assembly model in which components of the pathway are pieced together from preexisting genes or operons (Fani *et al.* 2005). This approach is very similar to that proposed for the pentachlorophenol (PCP) degradation pathway of *S. chlorophenolicum* L1, which shares some downstream components of the *lin* pathway. *S. chlorophenolicum* L1 was found to share the downstream pathway (homologs of the *linF* and *linEb* genes) with a common ancestor and to then have acquired three upstream genes responsible for the specific degradation of PCP through two separate horizontal transfer events (Copley *et al.* 2012). One of the transfer events brought in two catabolic genes in a regulated operon (similar to the case of *linRED*) and one brought in a single catabolic gene (similar to the proposed acquisition of *linA*, *linB*, and *linC*). In the case of the PCP pathway, however, the lack of a known mechanism for the horizontal transfer precludes a discussion of the future evolutionary trajectory of the system.

In the case of the *lin* system, however, we can identify some of the mechanisms involved and their continuing effects. Various methods of horizontal transfer have been responsible for the initial recruitment of the upstream *lin* genes to strains already possessing a downstream component (Nagata *et al.* 2014) and this work implicates an ICE in the acquisition of *linB*. An initial association of IS*6100* with some of the *lin* genes has spread such that the entire pathway (at least for the strains isolated from the Czech Republic) is now associated with IS*6100* elements. The *lin* pathway therefore provides valuable insight into the intermediate steps of IS*6100* activity, which has not been possible in previous investigations of IS*6100*-associated catabolic pathways. For example, in both the carbazole degradation pathway of *Sphingomonas* sp. XLDN2-5 and the 2-chloronitrobenzene degradation pathway of *Pseudomonas stutzeri* ZWLR2-1, the entire degradation pathway is organized into several large clusters flanked very closely by IS*6100* elements (Gai *et al.* 2010; Liu *et al.* 2011). Although the origins of these clusters have been identified, no instances of intermediate pathway assembly have been observed. Here, we show the dynamic nature of IS*6100*-mediated pathway assembly, with many different insertion sites around the *lin* genes. This suggests that the *lin* pathway is still in the process of evolving and that IS*6100* is playing an active role.

IS*6100* activity also may be resulting in a clustering of the *lin* genes. Homologous recombination between directly repeated IS*6100* elements can cause the deletion of the intervening genomic sequence (Mahillon and Chandler 1998). Although this is known to be responsible for deletions of *lin* genes (Nagata *et al.* 2011) it could also be responsible for bringing together upstream components of the pathway. In B90A we find a *linA1-linF-linC* cluster, and the *linF-linC* component also is found in IP26 and MM1-pISP1. In LL01 *linB* and *linC* are colocated, whereas HDIP04 and MM1-pISP4 each contain a *linB-linC-linF* cluster. This clustering is predicted by the Selfish Operon Theory, which posits that operon formation can be driven by selective pressure to reduce the number of horizontal transfer events required to acquire the pathway (Lawrence and Roth 1996). Although the contribution of this process to the formation of operons is disputed (Pál and Hurst 2004), the proposal that clustering of genes in the same pathway can result from horizontal transfer is supported by our observations of the *lin* pathway. Under this model, we expect to see additional clustering of the *lin* genes as strains continue to be exposed to HCH. Further sequencing of recently isolated strains will help determine if this predicted evolutionary trajectory is accurate.

## Supplementary Material

Supporting Information
