# Supplementary material for: Insights into Ongoing Evolution of the Hexachlorocyclohexane Catabolic Pathway from Comparative Genomics of Ten Sphingomonadaceae Strains
Source: G3 (Bethesda). 2015 Apr 7;5(6):1081–94. doi: 10.1534/g3.114.015933 (PMC4478539; doi:10.1534/g3.114.015933)
Supplement: Supporting Information [file supp_5_6_1081__index.html]

Insights into Ongoing Evolution of the Hexachlorocyclohexane Catabolic Pathway from Comparative Genomics of Ten Sphingomonadaceae Strains — Supporting Information 

# Insights into Ongoing Evolution of the Hexachlorocyclohexane Catabolic Pathway from Comparative Genomics of Ten Sphingomonadaceae Strains

## Supporting Information for Pearce, Oakeshott, and Pandey, 2015

**Files in this Data Supplement:**

- Supporting Information - Figures S1-S2 and Table S1 (PDF, 275 KB)
- Figure S1 - Summary of HCH isomer degradation. (PDF, 134 KB)
- Figure S2 - Genomic organization of *linKLMN*. (PDF, 185 KB)
- Table S1 - *lin* genes in *S. czechense* LL01, *N. barchiamii* LL02 and *S. baderi* LL03. (.xlsx, 11 KB)
