# Supplementary material for: Insights into Ongoing Evolution of the Hexachlorocyclohexane Catabolic Pathway from Comparative Genomics of Ten Sphingomonadaceae Strains
Source: G3 (Bethesda). 2015 Apr 7;5(6):1081–94. doi: 10.1534/g3.114.015933 (PMC4478539; doi:10.1534/g3.114.015933)
Supplement: Supporting Information [file supp_g3.114.015933_015933SI.pdf]

G3 · Genes | Genomes | Genetics

Insights into Ongoing Evolution of the Hexachlorocyclohexane Catabolic Pathway from Comparative Genomics of Ten Sphingomonadaceae Strains

Running title: Evolution of the HCH Catabolic Pathway

Stephen L Pearce, John G Oakeshott and Gunjan Pandey[#]

CSIRO Ecosystem Sciences, Clunies Ross Street (GPO Box 1700), Acton, ACT-2601, Australia

[#]**Corresponding author**: Tel: +61 2 62464244; Email: gunjan.pandey@csiro.au

DOI: 10.1534/g3.114.015933

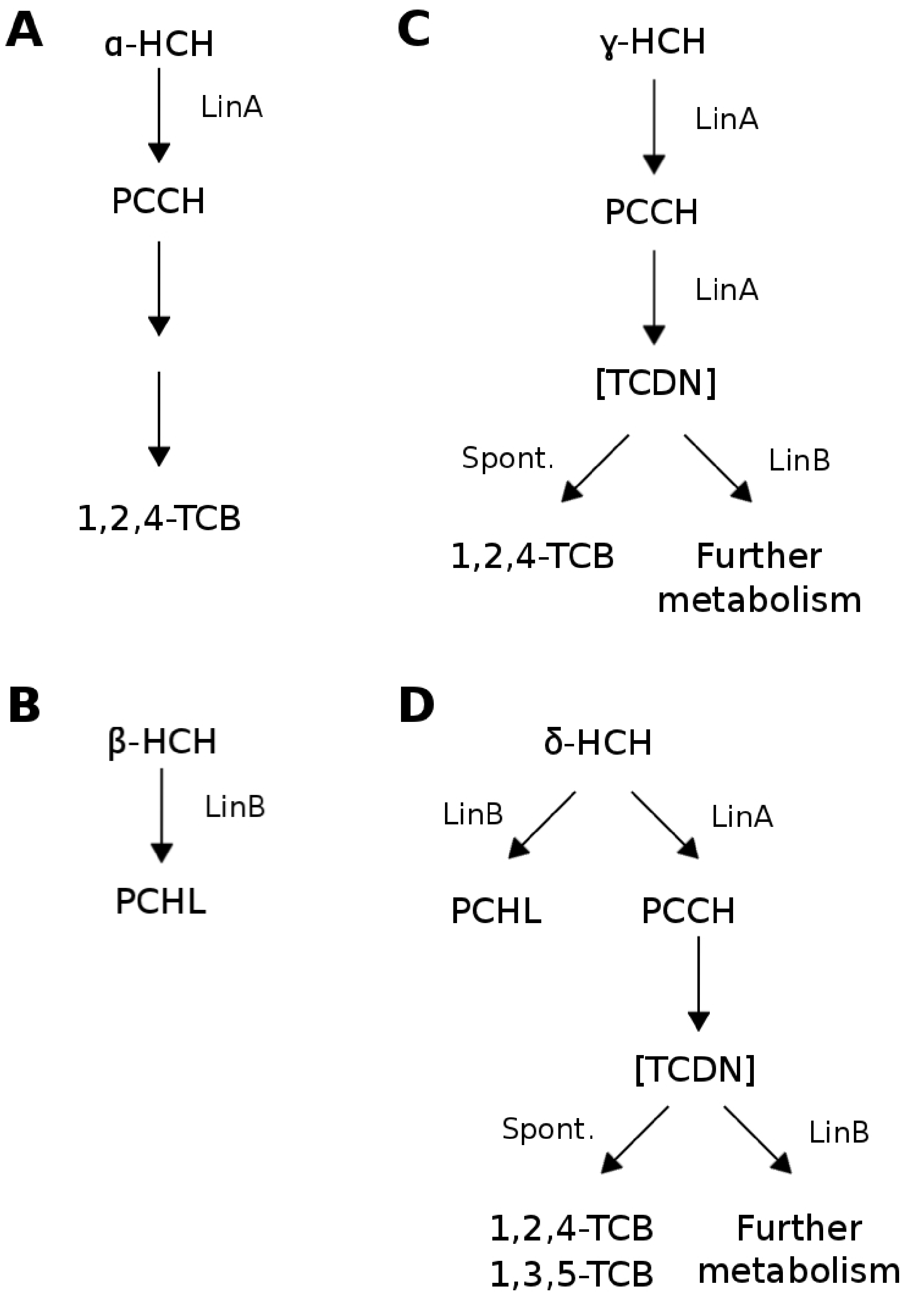

Figure S1 Summary of HCH isomer degradation. Simplified summary of the initial steps of (A) α-HCH, (B) β-HCH, (C) γ-HCH and (D) δ-HCH isomer degradation by LinA and LinB in UT26. Full pathways are described in reviews by Nagata *et al.* (2007) and Lal *et al.* (2010)

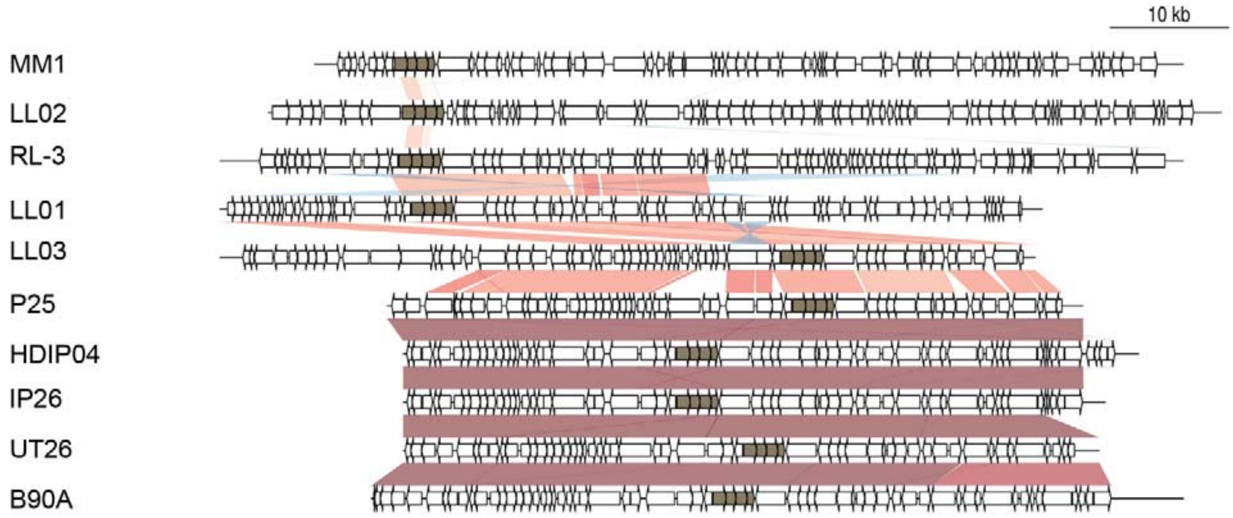

Figure S2 Genomic organization of *linKLMN*. Alignments detailing conservation and rearrangements in the genomic regions of the *linKLMN* genes. Alignments of the regions surrounding *linKLMN* (brown arrows) were performed with MEGABLAST and ordered to give maximal pairwise alignment lengths. Conserved regions are indicated by red shading where the matches are in the same orientation and by blue shading where the matches are in reverse orientation. Lighter shades indicate lower levels of identity.

Table S1 ***lin* genes in *S. czechense* LL01, *N. barchiamii* LL02 and *S. baderi* LL03**

Identification of each *lin* gene identified in *S. czechense* LL01, *N. barchiamii* LL02 and *S. baderi* LL03 along with encoded protein identity and coverage relative to the UT26 protein.

Available for download as an Excel file at www.g3journal.org/lookup/suppl/doi:10.1534/g3.114.015933/-/DC1
