# Supplementary material for: Insights into Ongoing Evolution of the Hexachlorocyclohexane Catabolic Pathway from Comparative Genomics of Ten Sphingomonadaceae Strains
Source: G3 (Bethesda). 2015 Apr 7;5(6):1081–94. doi: 10.1534/g3.114.015933 (PMC4478539; doi:10.1534/g3.114.015933)
Supplement: Supporting Information [file supp_g3.114.015933_FigureS1.pdf]

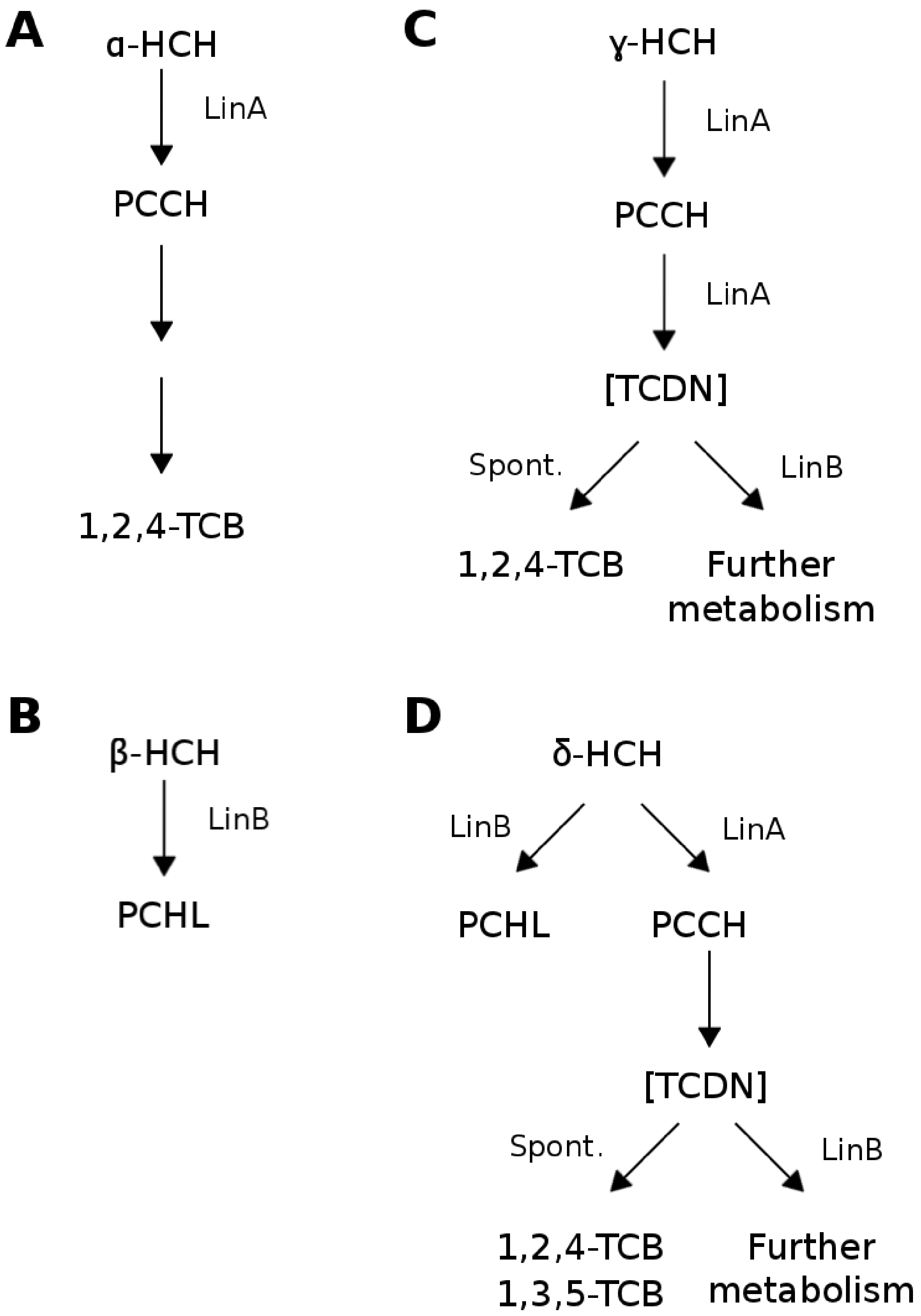

Figure S1 Summary of HCH isomer degradation. Simplified summary of the initial steps of (A) α-HCH, (B) β-HCH, (C) γ-HCH and (D) δ-HCH isomer degradation by LinA and LinB in UT26. Full pathways are described in reviews by Nagata *et al.* (2007) and Lal *et al.* (2010)
