# Supplementary material for: Insights into Ongoing Evolution of the Hexachlorocyclohexane Catabolic Pathway from Comparative Genomics of Ten Sphingomonadaceae Strains
Source: G3 (Bethesda). 2015 Apr 7;5(6):1081–94. doi: 10.1534/g3.114.015933 (PMC4478539; doi:10.1534/g3.114.015933)
Supplement: Supporting Information [file supp_g3.114.015933_FigureS2.pdf]

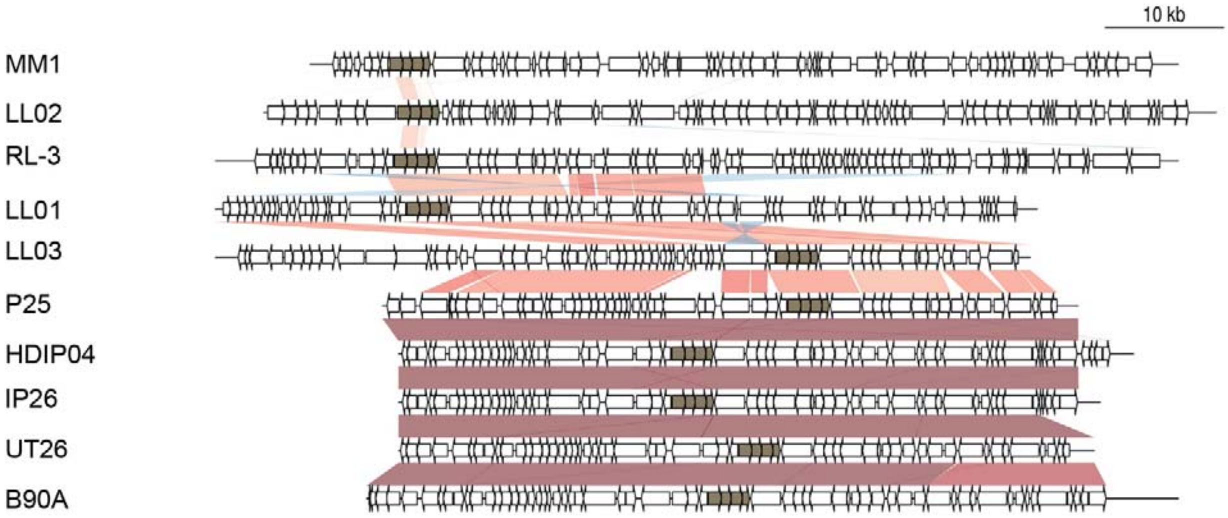

Figure S2 Genomic organization of *linKLMN*. Alignments detailing conservation and rearrangements in the genomic regions of the *linKLMN* genes. Alignments of the regions surrounding *linKLMN* (brown arrows) were performed with MEGABLAST and ordered to give maximal pairwise alignment lengths. Conserved regions are indicated by red shading where the matches are in the same orientation and by blue shading where the matches are in reverse orientation. Lighter shades indicate lower levels of identity.
